# Neuroprotective Effects of Casein-Derived Peptide Met-Lys-Pro (MKP) in a Hypertensive Model

**DOI:** 10.3389/fnins.2020.00845

**Published:** 2020-08-14

**Authors:** Asuka Matsuzaki Tada, Hamizah Shahirah Hamezah, Daijiro Yanagisawa, Shigehiro Morikawa, Ikuo Tooyama

**Affiliations:** ^1^Molecular Neuroscience Research Center, Shiga University of Medical Science, Otsu, Japan; ^2^Functional Food Ingredients Group, Food Ingredients and Technology Institute, R&D Division, Morinaga Milk Industry Co., Ltd., Zama, Japan

**Keywords:** brain, hypertension, ACE inhibitor, peptide, Met-Lys-Pro, MKP

## Abstract

We have previously reported that casein hydrolysate, CH-3, from bovine milk and casein-derived tripeptide Met-Lys-Pro (MKP) has ACE inhibitory activity and reduces blood pressure. In this study, we investigated the therapeutic effects of MKP in a hypertensive rat model (7-week-old male SHRSP/Izm rats). For long term evaluation, rats were fed either a diet containing CH-3 or normal diet. The survival rate of SHRSP rats was significantly improved by intake of CH-3 for 181 days. For short term evaluation, rats were orally administered synthetic tripeptide MKP or distilled water for 4 weeks. MRI study demonstrated that hemorrhagic lesions were observed in two of five rats in the control group, while no hemorrhagic lesions were observed in the MKP group. Volumetric analysis using MRI revealed that MKP administration inhibited atrophy of diencephalic regions. Histological examinations revealed that hemorrhage areas and astrogliosis in the hippocampus and cerebral cortex were lower in the MKP group than in the control group. Gene expression analysis indicated that MKP administration reduced expression of genes related to cerebral circulation insufficiency such as immune responses (*Cd74* and *Prkcd*), response to hypoxia (*Ddit4*, *Apold1*, and *Prkcd*), reactive oxygen species metabolic process (*Ddit4* and *Pdk4*), and apoptotic process (*Ddit4*, *Prkcd*, and *Sgk1*), suggesting that MKP administration prevented cerebral ischemia associated with hypertension. In addition, some genes encoding responses to hormone stimulus (*Fos*, *Dusp1*, and *Sik1*) were also downregulated. Serum aldosterone and corticosterone levels were also significantly decreased following MKP administration. The present study indicates that MKP shows neuroprotective effects in SHRSP rats by regulating cerebral circulation insufficiency and corticoid levels. MKP administration may therefore be a potential therapeutic strategy for hypertensive brain diseases such as cerebrovascular disease.

## Introduction

Hypertension is a major risk factor for cerebrovascular disorders such as stroke, vascular dementia, transient ischemic attacks, and subarachnoid hemorrhage. Elevated blood pressure (BP) induces arteriosclerosis in cerebral and peripheral blood vessels, and arteriosclerosis leads to a wide variety of diseases including ischemia, bleeding, and infarction (Fisher, 1965) as well as dementia (Walker et al., 2019). The incidence of stroke increases in proportion to both systolic and diastolic blood pressure, with a relative risk increase of 3.1 times in men and 2.9 times in women (Kannel et al., 1970, 1981). Hypertension is also known to exacerbate stroke outcomes. Although hypotension is also detrimental to stroke, hypertensive patients have larger infarcts and less peripheral tissue which can be rescued compared to normotensive patients (Leonardi-Bee et al., 2002; Ahmed et al., 2009). In addition, hypertension has been reported to be a major risk factor for cerebral small vessel disease (CSVD), which causes lacunar stroke and cognitive decline (Veglio et al., 2009). Several studies using a hypertensive model reported that vascular injury in the large and small blood vessels of the brain markedly reduced cerebral circulation and is involved in the exacerbation of stroke (Strandgaard, 1978; Muller et al., 2012). Circulatory failure in the brain occurs after inward remodeling in the aorta and small arteries, reducing the vascular lumen diameter and vasodilator reserve. It has been reported that ischemic damage due to cerebral circulatory failure may cause disruption of nerve activation, alteration of vasoactive metabolites, and vascular dysfunction (Faraci and Heistad, 1990). Thus, the influence of hypertension on cerebral circulation is considered important in the process of cerebrovascular disease.

Cerebral ischemia induces various pathological conditions in the brain such as hypoxia, oxidative stress, and inflammation. These pathological changes cause damage to neurons, glia, and vascular cells in the neurovascular unit as well as breakdown of the blood-brain barrier (BBB) through disengagement of tight junctions (Ohmori et al., 2018). Increased reactive oxygen species (ROS) induced extracellular matrix denaturation via matrix metalloproteinase (Rempe et al., 2016), and enhanced DNA damage that led to cell damage and apoptosis (Chan, 2001). ROS and fatty acid peroxides are also increased in neurodegenerative diseases, including AD, Parkinson’s disease, and amyotrophic lateral sclerosis. These findings suggest that oxidative stress impairs cellular functions and contributes to an exacerbation of neuropathology (Barnham et al., 2004; Singh et al., 2019). Wang and Xu (2020) reported that brain damage was suppressed by treatment with antioxidants such as curcumin. Since ischemic stress associated with arteriosclerosis triggers ROS production in cases of hypertension, antihypertensive agents such as angiotensin-converting enzyme (ACE) inhibitors and angiotensin II type 1 receptor blockers (ARBs) may be useful for early intervention.

The renin angiotensin system (RAS) is a hormone system within the body that is essential for the regulation of blood pressure and fluid balance. Angiotensin II is an important hormone of the RAS that causes endothelial dysfunction and has been shown to increase cerebral artery tone through its interaction with the angiotensin receptor 1 (AT1R) (Saavedra and Nishimura, 1999; Griendling et al., 2000; Dzau, 2001; Nickenig and Harrison, 2002). In addition, angiotensin II activated the NADPH-oxidase complex via AT1R and induced oxidative stress and inflammation (Labandeira-Garcia et al., 2017). It was recently reported that the RAS existed not only systemically, but also locally in organs such as the kidney and brain (Hosomi et al., 2013; Sansoè et al., 2020). Regulation of the RAS by ACE inhibitors and ARBs has been reported to improve stroke outcome in hypertensive animals and reduce the severity of ischemic stroke in humans (Werner et al., 1991; Walther et al., 2002; Iwai et al., 2004; Hosomi et al., 2005; Chitravas et al., 2007; Nagai et al., 2011; Panahpour et al., 2014). Several studies reported that regulation of the RAS by ACE inhibitors and ARBs has a cerebral protective effect against ischemic stroke independent of a hypotensive effect, whereas non-RAS antihypertensive agents such as hydralazine and nimodipine have not shown cerebral protection (Hosomi et al., 2005; Kozak et al., 2008; Omura-Matsuoka et al., 2009; Smeda and Daneshtalab, 2011; Smeda et al., 2018). Interestingly, these findings suggest that the beneficial effects of intervention to the renin-angiotensin system on ischemic stroke are not necessarily due to its antihypertensive effects. Furthermore, it has been reported that administration of ACE inhibitor captopril in Stroke-Prone Spontaneously Hypertensive Rat (SHRSP) exerted a tissue protective effect against stroke and created severe vascular lesions independently of the blood pressure lowering (Stier et al., 1991). Ohta et al. (1995) showed that the occurrence of vascular lesions and vascular necrosis were significantly suppressed in various organs, including the brain, in SHRSP rats treated with drugs (ACE inhibitor and calcium channel blockers), in doses which did not show antihypertensive effects, and blood pressure exceeded 250 mmHg for a long time. Although there was no difference in antihypertensive effects comparing ACE inhibitor and calcium channel blockers, inhibition of vascular smooth muscle cell proliferation was superior with ACE inhibitor. Regulation of RAS has been shown to reduce stroke-induced Ang II and matrix metalloproteinase activation, and one mechanism by which regulation of RAS improves outcome is to prevent edema formation and hemorrhagic transformation. It is suggested that these effects are due to suppression of degeneration of vascular smooth muscle cells (Ohta et al., 1995; Hosomi et al., 2005). Namely, the beneficial effects elicited by RAS modulation on stroke outcome is multifactorial. RAS modulation is highly useful because it can be expected to have complex physiological activities in addition to its known action via hypotension.

A number of studies have reported that food-derived components have antihypertensive effects. For example, food-derived peptides from milk (Ile-Pro-Pro and Val-Pro-Pro) and sardine peptides exert hypotensive effects via ACE inhibitory activity (Matsui et al., 1993; Nakamura et al., 1995). We also demonstrated that 5 mg/kg to 100 mg/kg of casein hydrolysate CH-3 has hypotensive effects in spontaneously hypertensive rats (SHRs) (Yamada et al., 2013). The functional component of CH-3 is Met-Lys-Pro (MKP), which is an αS2 casein-derived tripeptide with high ACE inhibitory activity (Yamada et al., 2013). Oral ingestion of 0.5 mg/kg to 1.0 mg/kg of MKP showed hypotensive effects for both single and multiple administrations in SHRs (Yamada et al., 2015). Our recent evidences have shown that MKP-containing peptides lowered systolic blood pressure in a clinical trial in subjects with high blood pressure (Yuda et al., 2018). However, these evaluation systems are relatively mild hypertensive models, and a model of severe hypertension has not yet been verified. In addition, animal studies suggested that MKP-containing peptides improved cognitive function and cerebral blood flow in an AD mouse model treated by amyloid β intracerebroventricular administration (Min et al., 2017). 250 mg/kg of CH-3 and 0.5 mg/kg of MKP reduced cognitive decline in this study. Further, autoradiography indicated that ^14^C-labeled MKP, Met-[1-^14^C]-Lys-Pro, was distributed in the brain, suggesting that MKP can cross the BBB to exert its effects (Min et al., 2017). However, the precise mechanism remains unknown. In this study, therefore, we explore the mechanisms underpinning the neuroprotective effects of MKP in a severe hypertensive model, SHRSP/Izm rats. This animal model shows extremely high blood pressure from young adulthood and easily develops cerebrovascular disorders, and has been widely used for evaluating cerebral vascular disorders and brain damage (Braun et al., 2012). Therefore, we used this model to conduct morphological and molecular biological evaluation to verify the brain protective effects of MKP.

The aim of this study is to clarify whether neuropathy in a severe hypertensive model is improved by MKP. This research is significant because elucidation of the function of MKP is expected to contribute to the prevention of cerebrovascular and related diseases associated with hypertension.

## Materials and Methods

### Animals

Male SHRSP/Izm at 6 weeks of age were used in the present study (Japan SLC, Hamamatsu, Japan). The rats were maintained in standard laboratory cages with free access to food and water throughout the study period under a 12 h light/dark cycle. The animals were acclimated for 1 week prior to the experiments and administration started at 7 weeks of age. CH-3 was prepared by Morinaga Milk Industry Co., Ltd. As described previously (Yamada et al., 2013), MKP content of CH-3 is 0.045%. MKP was prepared by solid-synthesis by Peptide Institute, Inc. (Ibaraki, Japan). All animal experimental procedures in this study were approved by the Committee on Animal Care and Use of Shiga University of Medical Science (Protocol #19-082 and #19-083) and the Institutional Animal Care and Use Committee of Morinaga Milk Industry Co., Ltd. (#16-043 and #18-071). The experimental period was from February 2017 to July 2019.

First, we conducted a study using CH-3 to observe whether food materials containing MKP are effective. To test the effects of CH-3 on survival rates in SHRSP/Izm rats, 20 rats were divided into two groups: 10 rats received a regular diet (SP diet; Funabashi Farm, Funabashi, Japan), and the other 10 rats received SP diet containing 0.1% CH-3. The supplementation was performed on 7-week-old rats. All animals were carefully monitored for 6 months. The average CH-3 intake during the administration period was 89 mg/kg.

To investigate the effects of MKP, the functional component of CH-3, on SHRSP/Izm rats, 20 rats were divided into two groups. Each group consisted of 10 rats that received MKP (0.5 mg/kg/day) or the vehicle (saline), once a day for 4 weeks by oral administration. The dose of MKP was determined with reference to previous studies (Yamada et al., 2015; Min et al., 2017). The rats were perfused through the aorta with 10 mM phosphate-buffered saline (pH 7.4; PBS) at 11 weeks of age under deep anesthesia with sevoflurane, and brains were quickly removed. Five of ten brains in each group were fixed with 4% paraformaldehyde (PFA) in 0.1 M phosphate buffer (pH 7.4; PB) for 24 h at 4°C, and then transferred to 15% sucrose in 0.1 M PB containing 0.1% sodium azide. The fixed brains were subjected to magnetic resonance imaging (MRI). The five remaining brains in each group were cut into two hemispheres in the sagittal plane. Half of the brain hemispheres were snap frozen and subjected to RNA extraction. The other half were immersed in 4% PFA in 0.1 M PB for 24 h at 4°C followed by 15% sucrose in 0.1 M PB containing 0.1% sodium azide for immunohistochemistry.

### Histological Analysis

Immunohistochemical analysis was performed as previously described (Yanagisawa et al., 2015; Ibrahim et al., 2017). Briefly, free-floating sagittal brain sections were washed with 0.1 M PBS containing 0.3% Triton X-100 (PBS-T, pH 7.4) and then incubated in 0.3% hydrogen peroxide in PBS-T for 20 min at room temperature (RT) to eliminate endogenous peroxidase activity. After three 10-min washes in PBS-T, the sections were blocked with 2% bovine serum albumin (BSA) in PBS-T for 30 min at RT. Blocked sections were then incubated with rabbit polyclonal anti-GFAP antibody (1:5,000; Dako, Glostrup, Denmark) in PBS-T containing 0.2% BSA overnight at 4°C. After several washes with PBS-T, immunolabeled sections were incubated with biotinylated goat polyclonal anti-rabbit IgG antibody (1:1,000; Vector Laboratories, Burlingame, CA, United States) for 60 min at RT. Following several washes with PBS-T, sections were incubated with avidin-biotin complex solution (Vectastain ABC Elite kit, 1:3,000; Vector Laboratories) for 60 min at RT, and washed several times with PBS-T. Immunolabeling was visualized with 3,3′-diaminobenzidine (Dojindo Laboratories, Kumamoto, Japan) with nickel ammonium sulfate. Images were captured with a camera (DP27, Olympus). GFAP-positive areas were measured with NIH ImageJ software. Images were transformed to eight-bit grayscale. Threshold was set to 120 for hippocampus and 170 for cortex and percent of pixel intensity was quantified. The value of GFAP-positive area in each animal was calculated by averaging the values from three sections collected every ten sections.

### Magnetic Resonance Imaging

*Ex vivo* MR data of fixed brains were acquired with a 7.0 T Unity Inova System (Agilent, Santa Clara, United States) (Amatsubo et al., 2009; Yanagisawa et al., 2011, 2014). A custom-made volume coil with a 3.5-cm inner diameter was used to collect the data. The MR images of rat brains were obtained with T_2_-weighted 3D fast spin echo sequence; 4,000-ms repetition time (TR), 20-ms effective echo time (TEeff), four echo train length with 10-ms echo spacing, a 24 mm × 24 mm × 16 mm field of view (FOV), and 256 × 128 × 96 acquisition matrices. The size of the acquisition pixel was 0.094 mm × 0.188 mm × 0.167 mm (2.93 nL). The total scan time of one data set was 3 h 24 min 56 sec. The acquired data were finally processed into a 3D image of 256 × 256 × 128 pixels using 3D Fourier transformation. Segmentation of gray matter (GM) and white matter (WM) was performed using tissue probability maps (TPMs). The voxel size was calculated with SPM software (Wellcome Trust Center for Neuroimaging, UCL Institute of Neurology, London, United Kingdom) (Hikishima et al., 2017). The rat label atlas was transformed into the brain morphology of each individual using advanced normalization tools (ANTs) software (Valdés-Hernández et al., 2011). The volume of the tissue segmentation was measured. To identify cerebral hemorrhage, itk-snap was used. The hemorrhage sites were defined as a region showing a clear low signal close to regions with a faint high signal intensity due to inflammation or edema on T_2_WI.

### Microarray Analysis

Total RNA in hippocampus was isolated using RNeasy Plus Universal Kit (Hilden, Germany) according to manufacturer’s instructions. RNA integrity, quality, and quantity were evaluated with microcapillary electrophoresis (2100 Bioanalyzer, Agilent Technologies, Santa Clara, CA, United States) using the RNA 6000 Nano kit. Only samples with an RNA integrity number (RIN) of greater than eight were used for further analysis. RNA samples (three from each group) were used for microarray experiment with SurePrint G3 Rat Gene Expression v2 8 × 60 K Microarrays according to the manufacturer’s instructions (Agilent Technologies, United States). The normalization and expression analysis of the microarray data were performed with GeneSpring GX software (v13.1, Agilent Technologies, United States). Normalized expression results were summarized as log2 transformed values for each transcript. Statistical analysis was performed with the linear analysis of microarray technique from “limma” package (Ritchie et al., 2015). Genes with significant changes in transcript abundance were selected based on two criteria: a *p*-value of less than 0.05 and a cutoff in transcript abundance of at least two-fold.

### RNA Preparation and Quantification of Gene Expression

Total RNA from animal tissue was isolated using RNeasy Plus Universal Kit according to the manufacturer’s protocol. The RNA concentration was determined spectrophotometrically, and cDNA was synthesized using the High Capacity cDNA Reverse Transcription Kit (Applied Biosystems, Foster City, United States). cDNA was amplified by real-time PCR with an Applied Biosystems 7500/7500 Fast Real-Time PCR System using SYBR Green fluorescence signals. Normalization for loading was accomplished by assessing *Gapdh*. The primer sets for target genes are shown in Table 1.

**TABLE 1:** The primers used in this study.

**Gene**	**Forward primer**	**Reverse primer**
Cd74	CCACTGTCCATGGATAACATGCTC	TGGGTAGTTCACGGGTCCAGA
Thbs2	GTATTCCCAAGGCCAGCACAG	CCAAATGCCCAAGTGAGACCTAA
Apold1	GAGAGCCATGGCCTTTGAGTTTA	GTCATTATCTCAGGACTTCGCAACA
Ddit4	GAACACGGCCAGCATTTCAG	AACCGAGTTTGGTTTGCTTTGAC
Nfkbia	TGACCATGGAAGTGATTGGTCAG	GATCACAGCCAAGTGGAGTGGA
Cd101	AGGGTGTCTGCCACCTCTAACCTA	GTTTCCAGCAGATCGGGACAA
Fos	AGCCGACTCCTTCTCCAGCA	AAGTTGGCACTAGAGACGGACAGAT
Fosl1	CTGCTAAGTGCAGAAACCGAAGA	GCTTCCAGCACCAGCTCAA
Fosl2	GTGAGCTGACGGAGAAGCTG	CTGCAGCTCAGCGATTTCTTT
Depp1	TGCCTTTGGATCAGACAATCTTTAC	CGAATCGTTGGCAAATGG
S100a11	AGATGCATCGAGTCCCTGATTG	GGAAATCTAGCTGCCCATCACTG
Sik1	CGGAGGCATACACTGGCTGA	CAGGACCTTCGCTTGCAGA
Dusp1	AATCCGGATGCAGCTCCTGTA	ACCCAAGGCGTCGAGCATA
Cel	CAAGATGGCTGGGTGTCTGAAG	CCATCGACGACAGGGATGAA
Pdk4	AACCCAAGCCACATTGGGAGTA	TCGCAGAGCATCTTTGCACAC
Sgk1	TGGGTGCCAAGGATGACT	CCTCAGTAAACTCGGGATCAAAG
Aspa	GGCTCCATTACCCTGCTCTGTTTA	CGTGATGCTGAGGACCAACTTCTA
Prkcd	CAAAGGCCGCTTCGAACTCTAC	GGCCATCCTTGTCCAGCATTAC
Gapdh	GGCACAGTCAAGGCTGAGAATG	ATGGTGGTGAAGACGCCAGTA

### LC-ESI-MS/MS for Serum Corticoid Measurement

Samples were prepared as described below to measure aldosterone (Ald) and corticosterone (B). As internal standards, aldosterone-d7 (Ald-d7) and corticosterone-d4 (B-d4) were added to the rat serum, which was diluted with 1 mL of distilled water. Steroids were then extracted by 4 mL of *t*-butyl methyl ether. After the organic layer was evaporated to dryness, the extract was dissolved in 0.5 mL of methanol and diluted with 1 mL of distilled water. The sample was applied to an Oasis MAX cartridge, which had been successively conditioned with 3 mL of methanol and 3 mL of distilled water. The cartridge was washed with 1 mL of 1% acetic acid and 1 mL of methanol/distilled water mixture (45:55, v/v) successively. The steroids were then eluted with 1 mL of methanol. After the eluted fraction was evaporated to dryness, the residue was reacted with 0.25 mL of hydrochloric acid/ethanol mixture (1:5, v/v) for 30 min at RT. The reaction mixture was neutralized with 1 mL of 5% sodium hydrogen carbonate solution and extracted with 4 mL of *t*-butyl methyl ether. After the organic layer was evaporated to dryness, the residue was reacted with 50 μL of the derivatization mixture (80 mg of 2-methyl-6-nitrobenzoic anhydride, 20 mg of 4-dimethylaminopyridine, and 40 mg of picolinic acid in 1 mL of acetonitrile) and 10 μL of triethylamine for 30 min at RT. After the reaction, the sample was diluted with 0.5 mL of ethyl acetate/hexane/acetic acid mixture (15:35:1, v/v/v), and the sample was applied to an InertSep SI cartridge, which had been successively conditioned with 3 mL of acetone and 3 mL of hexane. The cartridge was washed with 1 mL of hexane and 2 mL of ethyl acetate/hexane mixture (3:7, v/v). The derivatized steroids were then eluted with 2.5 mL of acetone/hexane mixture (7:3, v/v). After evaporation, the residue was dissolved in 0.1 mL of acetonitrile/distilled water mixture (2:3, v/v), and 20 μL of the solution was subjected to the Liquid Chromatograph – Mass Spectrometry (LC-MS/MS) instrument.

For measuring Ald and B in rat serum, an API-4000 triple stage quadrupole mass spectrometer (Sciex, Mass, United States) connected to LC-30AD, RACKCHANGER II (Shimadzu, Kyoto, Japan), ESI ion source device was employed. The column was a Kinetex C18 (150 mm × 2.1 mm, 1.7 μm, Phenomenex, CA, United States) used at 50°C. The mobile phase consisting of acetonitrile and 0.1% formic acid was used with a gradient elution at a flow rate of 0.5 mL/min. Ion spray voltage (IS), Collision Activated Dissociation (CAD), Curtain Gas (CUR), and ion source temperature (TEM) parameters were 5,500 V, 4 psi, 15 psi, and 350°C, respectively. The SRM transitions of Ald, Ald-d7, B, and B-d4 were *m/z* 494.2/448.3, 501.2/455.2, 452.3/311.3, and 456.3/315.3, respectively.

### Statistical Analysis

Differences between treatment groups were analyzed using SPSS 25.0 for Windows (SPSS Inc., Chicago, IL, United States). Data are expressed as mean ± SEM. A *p*-value of <0.05 was considered statistically significant. Student’s unpaired *t*-test or Mann-Whitney *U*-test were used for between-group comparisons. Survival rates were examined using the Kaplan-Meier method and compared using a log-rank test. Statistical analysis for microarrays is described in “Microarray Analysis” section.

## Results

### CH-3 Prolongs Life Span in SHRSP/Izm Rats

**FIGURE 1:**
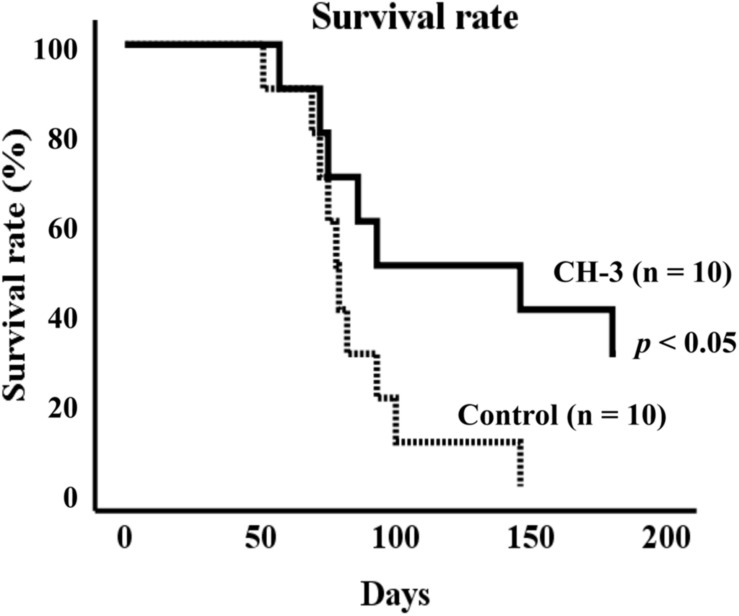
Survival rate of SHRSP/Izm rats up to 181 days. Survival curves were generated using Kaplan-Meier methods. Data were analyzed by log-rank test to compare groups. *n* = 10 per group.

To explore the effects of casein hydrolysate (CH-3) on life span, SHRSP/Izm rats were fed a SP diet with or without CH-3 for 181 days. Compared using the log-rank test, the survival rate was significantly improved in the CH-3 group (*p* < 0.05). In the control group, the mean life span was 85 ± 8 d during the experimental period. Mean life span was prolonged in the CH-3-treated group (125 ± 16 d). The CH-3 treatment resulted in a reduction of total mortality by 40% at the end of the experimental period (Figure 1).

### General Characteristics of Animals

**FIGURE 2:**
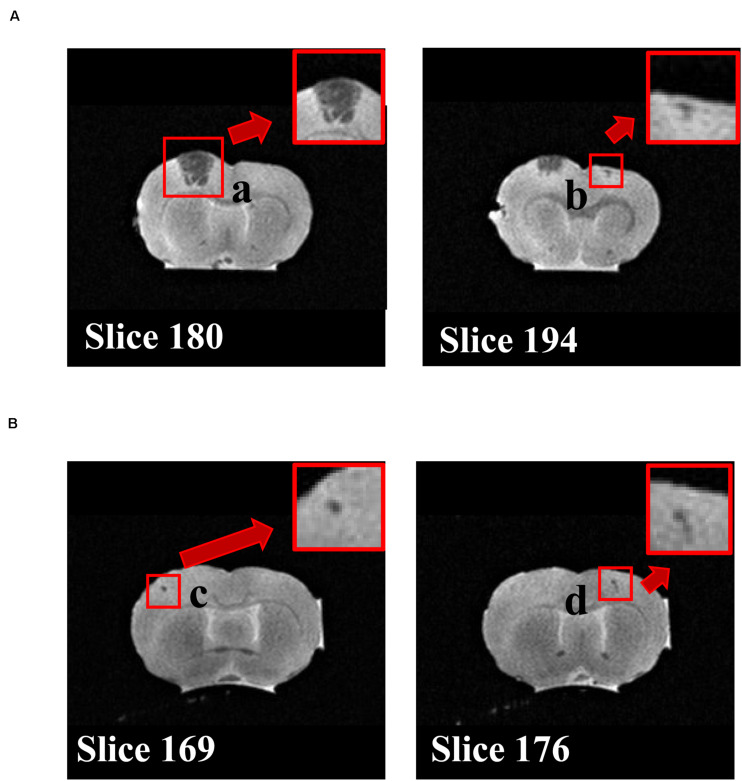
T_2_WI coronal MRI. The images of MRI slices in the rat indicate hemorrhage legions. Two individuals in the control group with lesions are shown in **(A)** and **(B)**, respectively. Cerebral hemorrhage (a–d) are depicted as regions showing a clear low signal near regions with a faint high signal intensity of inflammation or edema on T_2_WI. a–d show different lesion, respectively.

To determine the brain protective effects of tripeptide MKP, rats were administrated MKP for 4 weeks. Body weight and blood pressure were measured during the experimental period. After sacrifice, brains were excised and analyzed. There were no significant differences between groups in body weight, brain weight, blood pressure, and changes of blood pressure from baseline (Tables 2, 3). Changes in systolic blood pressure (SBP) over 4 weeks in the control and MKP groups were 65.7 ± 6.3 mmHg and 55.7 ± 4.8 mmHg, respectively. Changes in blood pressure showed a trend to be smaller in the MKP group but this did not reach significance. Hemorrhagic lesions in the brains were observed by *ex vivo* MR imaging in T_2_WI. In the MKP group, hemorrhagic lesions were not observed. In contrast, hemorrhagic lesions were observed in two of five rats in the control group (Figure 2, arrows). The volume of each hemorrhagic lesion was 11.13, 0.08, 0.07, and 0.04 mm^3^ for lesions a–d, respectively.

**TABLE 2:** Body and brain weights in SHRSP rats.

	**Body weight (g)**	**Brain (g)**	**Brain (g/kg BW)**
Control	257.87 ± 6.54	1.95 ± 0.02	7.59 ± 0.21
MKP	263.70 ± 3.23	1.95 ± 0.01	7.41 ± 0.09

*Values are presented as mean ± SEM. *n* = 9 in each group.*

**TABLE 3:** Changes in blood pressure from baseline in SHRSP rats.

	**SBP (mmHg)**		**DBP (mmHg)**	
	**2w**	**4w**	**2w**	**4w**
Control	41.3 ± 3.9	65.7 ± 6.3	30.1 ± 6.0	53.3 ± 7.4
MKP	35.4 ± 2.8	55.7 ± 4.8	34.0 ± 10.4	48.4 ± 7.3

*Values are presented as mean ± SEM. *n* = 9 in each group.*

### Immunohistochemical Staining of Brain Astrocytes

**FIGURE 3:**
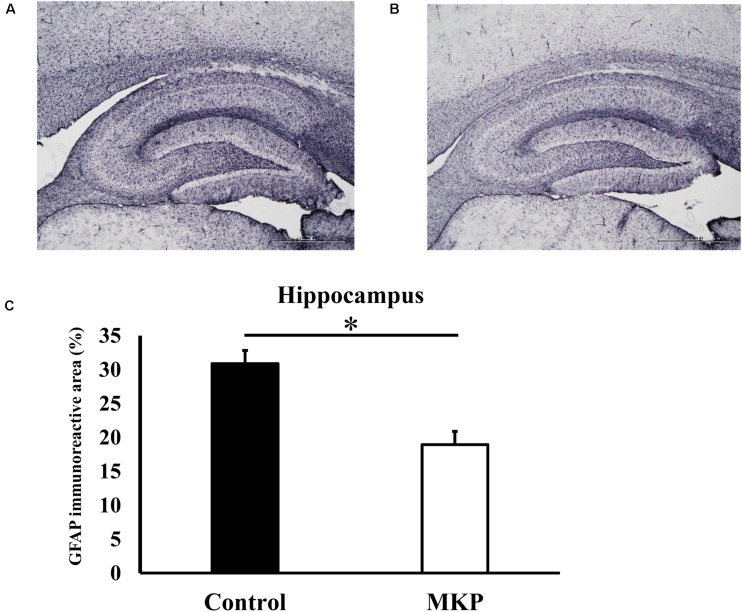
Expression of GFAP in the hippocampus. Immunohistochemistry images of control **(A)** and MKP **(B)** are shown. Percentage of immunoreactive area in each group is represented in **(C)**. Values are presented as mean ± SEM. Data were analyzed by Student’s *t-*test. *p < 0.05 compared to control group (Control: *n* = 4, MKP: *n* = 5).

**FIGURE 4:**
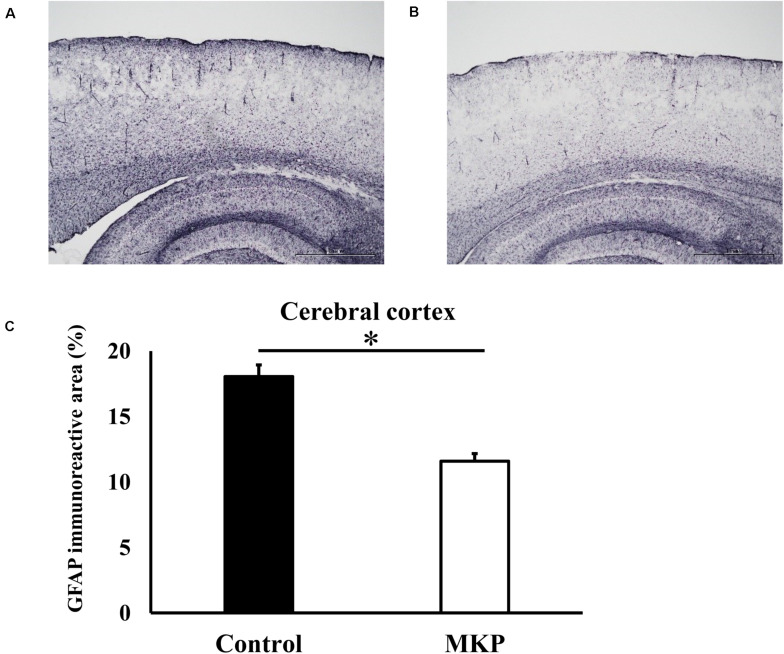
Expression of GFAP in the cerebral cortex. Immunohistochemistry images of control **(A)** and MKP **(B)** groups are shown. Percentage of immunoreactive area in each group is represented in **(C)**. Values are presented as mean ± SEM. Data were analyzed by Student’s *t-*test. *p < 0.05 compared to control group (Control: *n* = 4, MKP: *n* = 5).

Immunohistochemical staining for GFAP was performed to evaluate astrocyte activation in brain sections of SHRSP rats. Representative immunohistochemical staining for GFAP in the hippocampus and cerebral cortex are shown in Figures 3, 4, respectively. In the hippocampus, GFAP-positive areas in the control and MKP groups were 30.9 ± 2.0 and 18.9 ± 2.0%, respectively (Figure 3C). In the cerebral cortex, GFAP-positive areas in the control and MKP groups were 18.1 ± 0.9 and 11.6 ± 0.6%, respectively (Figure 4C). The MKP-treated group exhibited significantly lower GFAP-immunoreactivity in both the hippocampus and cerebral cortex compared to that in control rats (*p* < 0.05), suggesting that administration of MKP suppressed the activation of astrocytes in the SHRSP rat brain.

### Volumetric Analysis of Brain MRI

**FIGURE 5:**
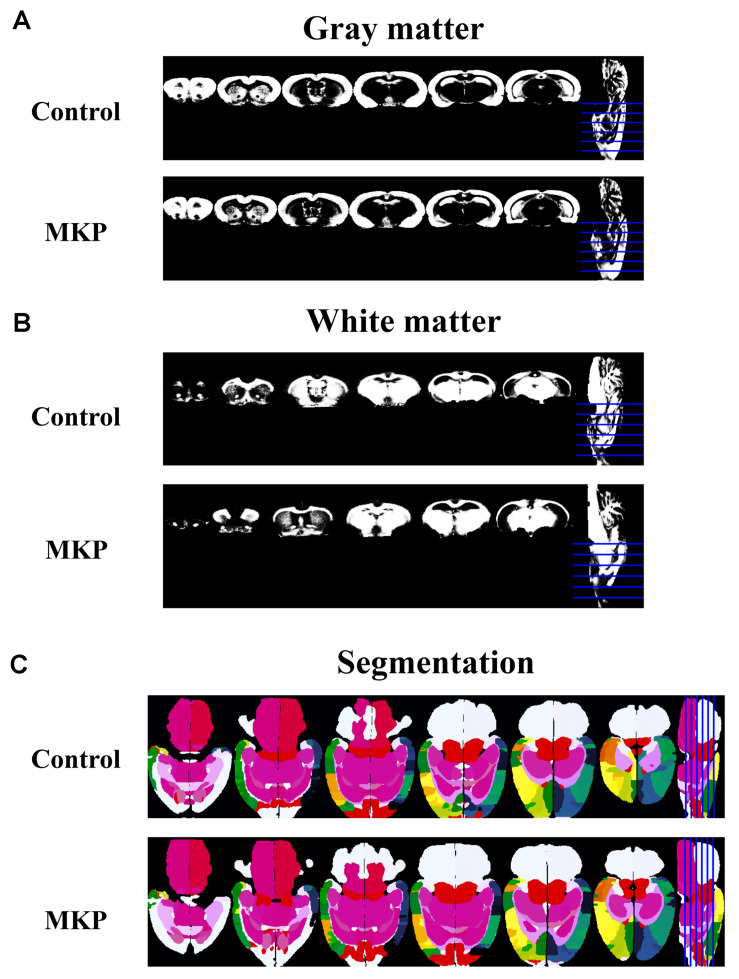
Coronal slices of gray matter and white matter detected using tissue probability maps (TPMs). MR images of gray matter **(A)** and white matter **(B)**. Horizontal slices of tissue segmentation slices are shown in **(C)**. The rat label atlas was transformed into the brain morphology of each individual using ANTs. The volume of each brain region was measured.

To evaluate brain morphological changes, volumetric analyses by MRI were performed. The total brain volume in control and MKP groups was 1751.4 ± 22.3 mm^3^ and 1774.9 ± 28.6 mm^3^, respectively. Next, we evaluated GM and WM volume. There was no difference in WM and GM volume between groups based on TPMs (Figures 5A,B and Table 4). Next, we conducted brain segmentation using a rat label atlas to evaluate the volume of a small region (Figure 5C). The volume of hippocampus and amygdala was not significantly different between groups. However, intergroup differences in volume changes were observed in the diencephalic region (*p* < 0.05). In the left hemisphere, the volume of the diencephalon (Control: 47.5 ± 0.6 mm^3^, MKP: 50.2 ± 0.5 mm^3^), pituitary (Control: 2.0 ± 0.1 mm^3^, MKP: 2.4 ± 0.1 mm^3^), and pallidum (Control: 7.8 ± 0.1 mm^3^, MKP: 8.2 ± 0.1 mm^3^) were significantly increased following MKP administration. The volume of the hypothalamus in the left (Control: 17.5 ± 0.3 mm^3^, MKP: 19.1 ± 0.5 mm^3^) and right hemispheres (Control: 17.9 ± 0.2 mm^3^, MKP: 19.5 ± 0.6 mm^3^) showed an increasing trend with MKP administration. However, statistical significance could not be claimed (Table 4).

**TABLE 4:** Volume analysis of tissue segmentation by MRI.

**Total**	**Control**	**MKP**
Brain total volume (mm^3^)	1751.4 ± 22.3	1774.9 ± 28.6
Gray matter (mm^3^)	858.0 ± 40.4	893.7 ± 58.5
White matter (mm^3^)	793.4 ± 37.4	778.1 ± 21.2
Gray matter (%)	49.1 ± 2.5	50.2 ± 2.6
White matter (%)	45.3 ± 1.8	44.0 ± 1.9

**Left hemisphere (mm^3^)**	**Control**	**MKP**

Hippocampal formation	52.1 ± 1.8	51.0 ± 0.4
Amygdala	19.6 ± 0.6	19.5 ± 0.6
Diencephalon	47.5 ± 0.6	50.2 ± 0.5*
Hypothalamus	17.5 ± 0.3	19.1 ± 0.5
Pituitary	2.0 ± 0.1	2.4 ± 0.1*
Striatum	40.7 ± 1.1	40.7 ± 0.4
Pallidum	7.8 ± 0.1	8.2 ± 0.1*
Substantia nigra	2.2 ± 0.2	2.8 ± 0.1

**Right hemisphere (mm^3^)**	**Control**	**MKP**

Hippocampal formation	55.3 ± 1.5	52.8 ± 0.9
Amygdala	21.5 ± 0.8	20.4 ± 0.7
Diencephalon	46.9 ± 0.6	48.5 ± 0.5
Hypothalamus	17.9 ± 0.2	19.5 ± 0.6
Pituitary	2.0 ± 0.1	2.4 ± 0.1
Striatum	39.5 ± 0.7	41.1 ± 1.3
Pallidum	8.5 ± 0.1	8.7 ± 0.3
Substantia nigra	2.2 ± 0.2	2.3 ± 0.1

*Data were analyzed by Mann-Whitney *U*-test. ^∗^*p* < 0.05 compared to control group (Control: *n* = 5, MKP: *n* = 4).*

### Hippocampus Transcriptomic Signature in SHRSP Rats

**FIGURE 6:**
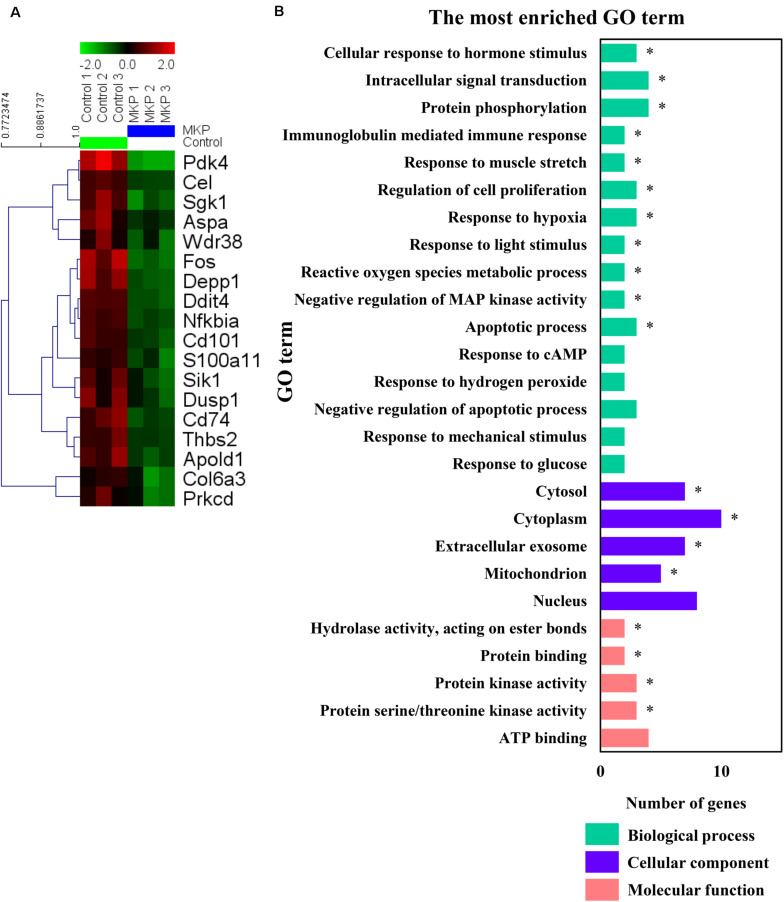
Microarray analysis in the hippocampus. **(A)** A heat map generated in MeV showing genes that were two-fold downregulated and significantly lower in limma analysis by administration of MKP. **(B)** GO term enrichment analysis of the downregulated genes. The vertical coordinates are the enriched GO terms, and the horizontal coordinates are the numbers of the downregulated genes in these GO terms. The green, purple, and orange columns represent the biological process GO terms, cellular component GO terms, and molecular function GO terms, respectively. *p-value < 0.05. The *p*-values were obtained from the DAVID 2.1 method of statistical function classification tool. *n* = 3 per group.

To identify early changes in hippocampal gene expression following MKP treatment in SHRSP rats, transcriptional profiles of hippocampal tissue treated with MKP were compared to control tissue by microarray. No genes were significantly upregulated by MKP administration compared to control. Conversely, *Ddit4, Cel, Nfkbia, Pdk4, Cd101, Fos, Depp1 Cd74, Sgk1, Thbs2, Apold1, S100a11, Sik1, Aspa, Dusp1, Col6a3, Wdr38*, and *Prkcd* were 0.5-fold downregulated compared to control levels (Figure 6A). Gene Ontology (GO) term enrichment analyses were performed on the 18 downregulated genes in the MKP treatment group with DAVID 6.8. We identified 11 significant gene terms in GO biological processes, four terms in GO cellular components, and four terms in GO molecular function (Figure 6B). Of note, identified genes were related to cerebral circulation insufficiency such as immune responses (*Cd74* and *Prkcd*), response to hypoxia (*Ddit4*, *Apold1*, and *Prkcd*), reactive oxygen species metabolic process (*Ddit4* and *Pdk4*) and apoptotic process (*Ddit4*, *Prkcd*, and *Sgk1*), suggesting that MKP administration prevented cerebral ischemia associated with hypertension. In addition, some genes encoding responses to hormone stimulus (*Fos*, *Dusp1*, and *Sik1*) were also downregulated.

### Validation of mRNA Expression

**FIGURE 7:**
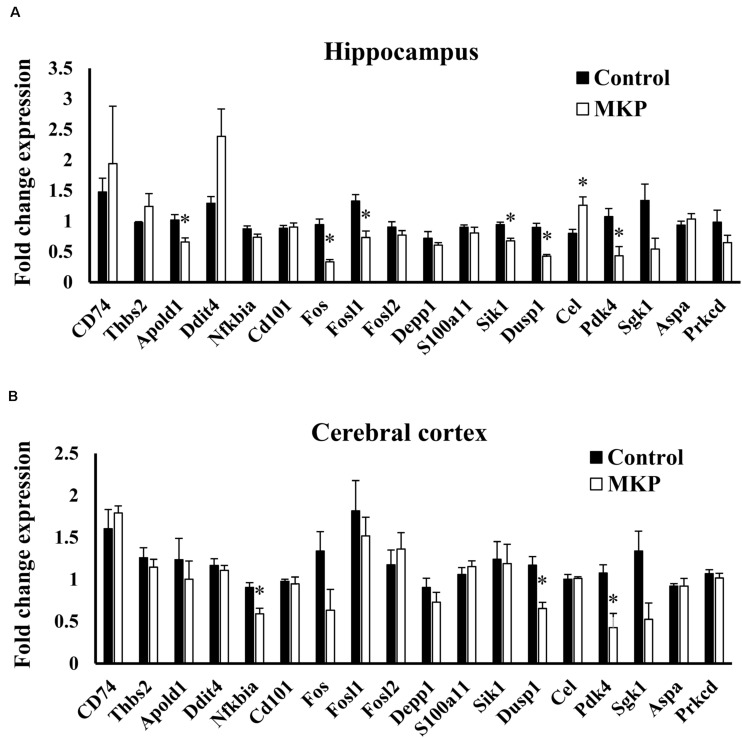
RT-qPCR validation of expression changes of selected genes in the hippocampus and cerebral cortex in SHRSP/Izm rats. The normalized fold expression of each gene in the hippocampus **(A)** and cerebral cortex **(B)** are represented in each bar as mean ± SEM. Data were analyzed by Student’s *t-*test. *p < 0.05 compared to control group (Control: *n* = 4, MKP: *n* = 5).

To confirm the expression of genes identified in microarray analysis, total RNA from the hippocampus and cerebral cortex was measured by RT-qPCR. *Apold1, Fos, Fosl1, Sik1, Dusp1*, and *Pdk4* were significantly downregulated by MKP administration in the hippocampus (*p* < 0.05), (Figure 7A). These genes were less than 0.65-fold decreased. *Cel* was 1.57-fold upregulated in the MKP group. In the cerebral cortex, *Nfkbia, Dusp1*, and *Pdk4* were significantly downregulated by MKP administration (*p* < 0.05), (Figure 7B). These genes were less than 0.70-fold decreased. *Fos, Dusp1*, and *Pdk4* encode cellular responses to hormonal stimuli. Other downregulated genes are involved in responses to hypoxia and ROS metabolism, indicating that oral intake of MKP suppressed cerebral circulatory insufficiency associated with hypertension and modulated hormonal responses in the brain.

### Serum Corticoids

**FIGURE 8:**
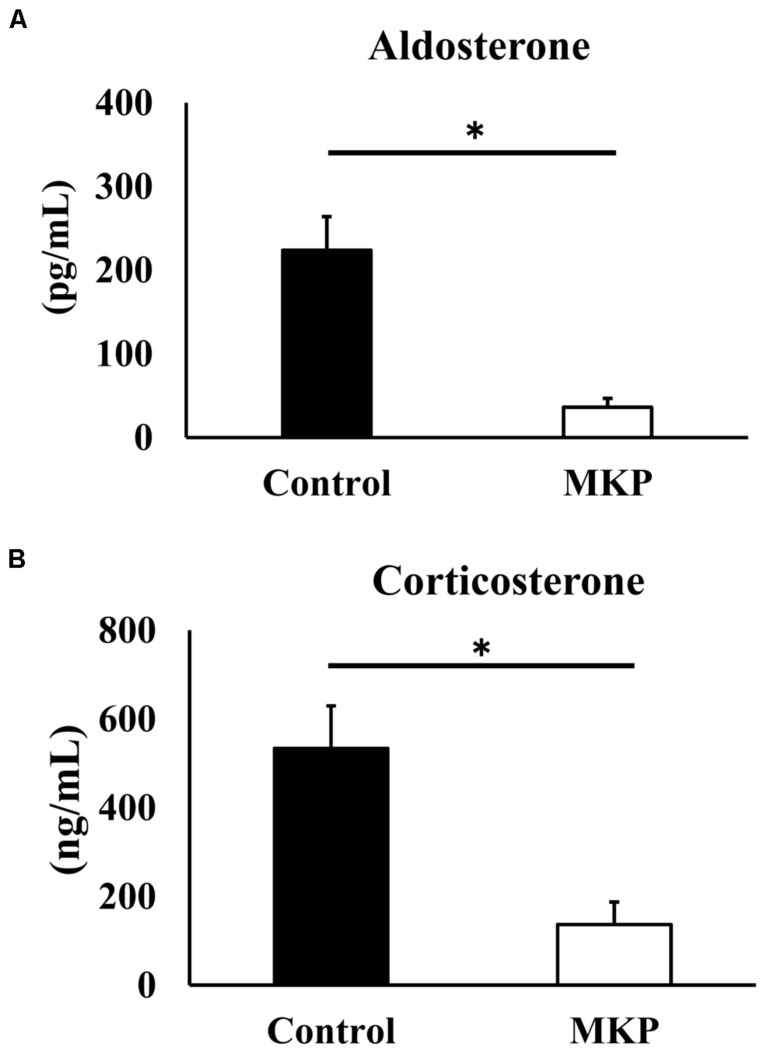
Serum corticoids in SHRSP/Izm rats. The concentration of aldosterone **(A)** and corticosterone **(B)** are represented in each bar as mean ± SEM. Data were analyzed by Student’s *t-*test. *p < 0.05 compared to control group (Control: *n* = 9, MKP: *n* = 9).

Based on the gene expression results, we hypothesized that metabolic changes in corticoids had occurred, so we measured serum mineralocorticoids (aldosterone) and glucocorticoid (corticosterone) levels. Serum aldosterone levels of control and MKP groups were 223.8 ± 40.4 pg/mL and 36.3 ± 10.4 pg/mL, respectively. Serum corticosterone levels of control and MKP groups were 534.2 ± 95.6 ng/mL and 136.8 ± 50.7 ng/mL, respectively. As such, MKP administration significantly suppressed the production of serum corticoids (*p* < 0.05) (Figure 8).

## Discussion

A previous report showed that intake of CH-3 or MKP reduced blood pressure in SHR rats (Yamada et al., 2013). In addition, oral intake of MKP suppressed the elevation of blood pressure in patients with high-normal blood pressure (Yuda et al., 2018). However, these models are relatively mild hypertension models, and the effects of MKP have not been examined in models that develop severe hypertension such as SHRSP. In this study, we first examined the survival rate using CH-3, casein hydrolysate, which contains MKP. We also demonstrated that the survival rate of SHRSP rats was significantly improved by intake of CH-3 for 181 days (Figure 1). Minami et al. (1985) have examined the cause of death in SHRSP, and it has been reported that all SHRSP rats die from cerebral hemorrhage or cerebral infarction. We estimated that cerebral hemorrhage and cerebral infarction were the causes of death in the present study as well. Since improvement in survival rate was observed in this experiment, the effect of MKP, which we are focusing on as an active ingredient of CH-3, was directly examined in following experiments. In the short-term study on MKP, the oral administration of MKP for 4 weeks showed a trend of smaller elevation of blood pressure in the MKP group than control group but was not significant (Table 3). Previous studies have reported that ACE inhibitors and ARBs administered to SHRSP rats produced brain-protective effects, such as suppression of cerebral hernia and hematoma expansion, without lowering blood pressure, indicating that the antihypertensive effect is not always required for brain-protective effects (Smeda and Daneshtalab, 2011; Smeda et al., 2018). However, we believe that the effect of MKP could be examined with a higher accuracy by reviewing the experimental methods, such as the dosage, based on the results of the present study, and we regard it as a future issue. The MRI study demonstrated that hemorrhagic lesions were observed in two of five rats in the control group, while no hemorrhagic lesions were observed in MKP group (Figure 2). In the previous study by Hayasaka et al. (2012), the same bleeding lesion as we found in our study was evaluated in SHRSP by various imaging methods such as contrast enhanced CT, T_2_WI and H&E staining, and based on their analysis we feel the evaluation of T_2_WI was appropriate. Taken together, not only CH-3 but also MKP have the capacity to reduce cerebral hemorrhage and prolong the lifespan.

Previously, we reported that MKP has ACE inhibitory activity and showed hypotensive effects (Yamada et al., 2013, 2015; Yuda et al., 2018). Therefore, we hypothesized that MKP exerted brain protective effects via lowering blood pressure in hypertensive model rats. However, MKP did not significantly decrease blood pressure of SHRSP rats used in this study. As SHRSP rats show high blood pressure values from a young age, it is assumed that ACE inhibitory effects of MKP were mild and did not produce statistically significant differences in lowered blood pressure. Our results suggest that MKP may affect the brain independently of changes in blood pressure. The hypothesis is supported by a previous report showing that antihypertensive drugs such as captopril inhibited hemorrhagic expansion through non-pressure related physiological changes in SHRSP rats (Smeda et al., 2018).

Several studies have reported that ischemia caused neuron loss and oxidative stress in the hippocampus in a hypertensive model (Sakurai-Yamashita et al., 2003; Solis-Gaspar et al., 2016). To examine the underlying mechanism of cerebral protective effects of MKP in SHRSP rats, we performed histochemical and molecular biological analyses. Several studies using GFAP-positive astrocytes as a marker of brain damage demonstrated that activation of astrocytes was increased in a mouse model of middle cerebral artery occlusion (MCAO) or in spontaneously hypertensive rats (Tayebati et al., 2009; Choudhury et al., 2014). The present study also showed that astrogliosis was seen in the hippocampus and cerebral cortex of SHRSP rats, and that activation of astrocytes in both regions was suppressed by ingestion of MKP (Figures 3, 4). Astrocytes are widely distributed in the mammalian central nervous system (CNS) and play an important role in maintaining nervous system homeostasis (Allen and Eroglu, 2017), contributing to maintenance of brain function via immune modulation and the BBB (Lo et al., 2003). GFAP is typically used to identify activated astrocytes within the CNS (Eng, 1985). Astrocytic proliferation has been reported in acute conditions such as cerebral ischemia, as well as neurodegenerative diseases such as AD and Parkinson’s disease (Liddelow et al., 2017). In addition, astrocytes have been reported to be involved in lesion repair by removing oxidative stress via the production of extracellular superoxide dismutase and protecting neurons via endogenous ketone body production (Puchowicz et al., 2008; Takano et al., 2013). Therefore, astrocytic proliferation has been used as an important pathological marker for stress. Here, our results suggest that MKP reduced pathological changes in the brain of SHRSP rats.

The volume of the whole brain was not different between the groups when measured by MRI as well as by tissue weight (Tables 2, 4). Besides, MRI experiments showed that GM and WM volumes were not different between MKP and control groups, but volumes of specific regions were significantly different. A preliminary evaluation of volumes of the diencephalon such as the pituitary showed they were increased compared to those in controls. In contrast, there was no difference in the volume of the hippocampus and amygdala (Table 4). Sakurai-Yamashita et al. (2003) reported that SHRSP rats with brain ischemic stress exhibited neuronal loss with morphological changes in the hippocampus. However, the present study did not show significant changes in the hippocampus. Taking the case of diencephalon region, Tan et al. (2017) reported that the hypopituitarism caused by traumatic brain injury (TBI) was primarily caused by decreased blood supply, and secondary damage caused by compression of the hypothalamus-pituitary was due to oxidative stress, cerebral edema and cerebral hemorrhage. An intracranial hypertension model displayed increased hippocampal, hypothalamic and pituitary apoptotic rates, but the hypothalamic and pituitary apoptotic rates were significantly higher than hippocampus (Tan et al., 2017). In addition, several studies have reported that cortical infarction causes non-ischemic apoptosis in ipsilateral thalamic and hippocampal neurons and may be associated with post-stroke cognitive impairment (Liao et al., 2013; Chen et al., 2017). These studies reported loss of neurons in regions such as the thalamus is a secondary injury of infarction in the cortex; it is possible that similar secondary injuries have occurred in the present study where some lesions in the cortex were observed. Furthermore, concerning the hypothalamus, a previous study reported that BBB permeability in the hypothalamus was significantly enhanced in SHRSP rats due to chronic hypertension (Ueno et al., 2004). The disruption of blood vessels is considered one of the causes of the damage to the hypothalamic tissues in the hypertensive model. It has been suggested that MKP may affect the volume of the diencephalic region by suppressing such brain tissue damage. It remains unclear whether the action is through suppression of secondary damage or of primary damage caused by BBB disruption and blood flow decreases in the diencephalic region. Analysis of the diencephalon region from the viewpoint of molecular biology and pathology is regarded as an important issue for the future.

The results of our microarray analysis of the hippocampus indicated that MKP administration downregulated the expression of genes related to immune responses, responses to hypoxia, and metabolism of ROS, which are related to cerebral vascular insufficiency due to cerebral arteriosclerosis. Notably, some genes encoding hormonal stimuli were also downregulated in the MKP group (Figure 6). To verify the expression of selected genes from microarray analysis, real-time RT-PCR analyses were performed. In the hippocampus, MKP administration significantly decreased the gene expression of *Apold1, Fos, Fosl1, Sik1, Dusp1*, and *Pdk4* (Figure 7A). In the cerebral cortex, MKP significantly decreased the gene expression of *Nfkbia, Dusp1*, and *Pdk4* (Figure 7B). Among them, *Fos, Dusp1*, and *Sik1* encode cellular responses to hormonal stimuli. Other downregulated genes were associated with responses to hypoxia and ROS metabolism. Therefore, the results suggest that MKP could inhibit arteriosclerosis and oxidative stress in the brain. Concerning genes related to cellular responses to hormonal stimuli (*Fos, Dusp1*, and *Sik1*), it was assumed that MKP affected the production of corticoids such as aldosterone via ACE inhibitory activity. Hence, we measured the concentration of serum corticoids. Both aldosterone and corticosterone levels were significantly decreased by MKP administration compared to those of controls.

Corticoids regulate glutamatergic synaptic transmission in the brain and affect learning and memory processes (Prager and Johnson, 2009). Mineralocorticoid and glucocorticoid overproduction induces brain nerve cell damage (Sherin and Nemeroff, 2011; Sasaki and Yoshizaki, 2015). Moreover, modulation of mineralocorticoid receptors (MRs) and glucocorticoid receptors (GRs) in the brain affects cognitive and emotional function (de Kloet et al., 2018; Wingenfeld and Otte, 2019). Brocca et al. (2019) reported that hippocampal damage in a hypertensive model was related to altered balance in the activity of MR and GR. They reported that oxidative stress caused by vascular damage and hypoxia promoted MR activation in the hippocampus. Several studies reported that serum aldosterone in Wistar rats was around 100 pg/mL, and corticosterone was around 140 ng/mL(Veyrat-Durebex et al., 2012; Svitok et al., 2014). In our study, we found that these parameters, which were high in SHRSP rats, were suppressed to normal levels in the MKP group. Our results revealed that the administration of MKP reduced serum corticoid levels (Figure 8). ACE inhibitory activity has been reported by MKP, and it is assumed that the effect on serum aldosterone is mediated by the regulation of RAS. The reduction of corticosteroids may alter expression of genes associated with MR and GR. Harvey et al. (2017) reported that aldosterone is increased in SHRSP, and vascular damage such as vascular fibrosis and inflammation is induced by activation of MR-Nox1-p66Shc signaling pathway. Reduction of vascular damage may be a factor by which MKP reduces oxidative stress and ischemic response in brain tissue. Since it has been indicated that cerebrovascular disease is involved in the pathogenesis of dementia, we consider this an important perspective to explore in future MKP research. Further, several studies reported that cognitive decline occurs in SHRSP (Kimura et al., 2000; Tang et al., 2015), thus we believe future behavioral pharmacological evaluations of this model will be useful. Equally important, changes in cortisol metabolism in the brain were not measured in this study. In addition, there were no changes in the expression of genes relating to the renin-angiotensin-aldosterone system. Further studies on MKP are needed to better understand the mechanisms of tissue protection of neurons and blood vessels, and the regulation of corticoids.

## Conclusion

The present study was the first to examine the effects on blood pressure and brain tissue by MKP in a severe hypertension model. Intake of MKP-containing casein hydrolysate CH-3 improved the survival rate of hypertensive rats. Although there was no significant difference in blood pressure, MKP suppressed symptoms of tissue damage in hypertensive models, such as cerebral hemorrhage, astrogliosis, and atrophy in the diencephalon region. In addition, gene expression analysis suggested that MKP administration improved cerebral circulation insufficiency and reduced oxidative stress. Furthermore, corticoid regulation by MKP may be involved in reducing damage in the brain. Our findings support the use of MKP as an intervention to protect the brain during hypertension, thereby contributing to the prevention of cerebrovascular disease.

## Data Availability Statement

The raw data supporting the conclusions of this article will be made available by the authors, without undue reservation.

## Ethics Statement

The animal study was reviewed and approved by The Committee on Animal Care and Use of Shiga University of Medical Science and the Institutional Animal Care and Use Committee of Morinaga Milk Industry Co., Ltd.

## Author Contributions

AT conceived and designed the project, performed all the experiments related to administration of MKP, and performed the image processing and analysis. AT, HH, and DY performed the experiments related to MR image acquisition under the supervision of SM. AT, DY, SM, and IT wrote and edited the manuscript. All authors contributed to the manuscript revision and read and approved the submitted version of the manuscript.

## Conflict of Interest

This study was funded by Morinaga Milk Industry Co., Ltd. AT is employed by Morinaga Milk Industry Co., Ltd. The remaining authors declare that the research was conducted in the absence of any commercial or financial relationships that could be construed as a potential conflict of interest.
